# Sepsis neonatorum: Bacterial profile, antimicrobial resistance patterns, among neonates and associated factors, Dilla University Referral Hospital, southern Ethiopia, with special reference to WHO prioritized pathogens

**DOI:** 10.1371/journal.pone.0352190

**Published:** 2026-07-10

**Authors:** Asaye Mitiku, Aseer Manilal, Eyerusalem Asnake, Bamud Hussien, Binyam Debalke, Alayu Bogale, Zerihun Solomon, Gelila Birsaw

**Affiliations:** 1 Department of Medical Laboratory Science, College of Medicine and Health Sciences, Dilla University, Dilla, Ethiopia; 2 Department of Medical Laboratory Science, Komar University of Science and Technology, Sulaymaniyah, Kurdistan region, Iraq; 3 Departments of Medical Laboratory Science, College of Medicine and Health Sciences, Arba Minch University, Arba Minch, Ethiopia; Hawassa University College of Medicine and Health Sciences, ETHIOPIA

## Abstract

**Background:**

Sepsis neonatorum (neonatal sepsis) constitutes severe, life-threatening infections, frequently necessitating intensive care unit admissions, and is a leading indication for antibiotic prescription in neonatal clinical practices. In Ethiopia, neonatal sepsis is quite rampant, and the widespread empirical therapy results in several interrelated challenges, such as incorrect or misuse of antibiotics, bacterial virulence, and aggravation of drug resistance and treatment failures.

**Aims:**

This study was done to assess the culture-positive sepsis, antibiotic susceptibility patterns, and associated factors among neonates attending Dilla University Referral Hospital, southern Ethiopia.

**Methods:**

A cross-sectional study was conducted from August to October 2023 among 280 neonates suspected of sepsis using systematic random sampling. Data and venous blood samples were collected and analyzed using standard microbiological procedures. Antibiotic susceptibility was tested by Kirby-Bauer disk diffusion. SPSS and logistic regression were used for analysis, with p ≤ 0.05 considered statistically significant.

**Results:**

The overall culture-positive prevalence (n = 88) of neonatal sepsis was 31.4% (95% CI: 25.7, 36.8). Neonatal sepsis was predominantly caused by *Escherichia coli* (n = 27, 30.7%), followed by *Klebsiella pneumoniae* (n = 18, 20.5%). Both Gram-negative and Gram-positive bacteria were extremely resistant (80–100%) to ampicillin, amoxicillin-clavulanate, ceftriaxone, and ceftazidime. Overall, 70.2% of the isolates were multi-drug resistant, including more than a quarter of isolates that were WHO prioritized, viz., methicillin-resistant *Staphylococcus aureus* and extended-spectrum β-lactamase bacteria. Isolates exceeding one-fourth of the entire bacteria studied demonstrated resistance to both the first- and second-line regimens. Residence in rural areas [AOR: 1.43; 95% CI: 1.22–5.84 (*p* < 0.05)], mode of delivery by Caesarean section [AOR: 3.23; 95% CI: 1.04–9.46 (*p* < 0.002)], lengthy hospital stays [AOR: 4.65; 95% CI: 1.55–13.9 (*p* < 0.001)], prolonged labour [AOR: 3.36; 95% CI: 1.32–8.56 (*p* < 0.05)], and preterm premature rupture of membrane [AOR: 5.23; 95% CI: 4.11–9.49 (*p* < 0.001)] were statistically associated with the outcome.

**Conclusions:**

A substantial prevalence of culture-confirmed cases of neonatal sepsis was identified, in comparison to other regions of the country. Alarmingly, bacterial isolates demonstrated considerable resistance to the first-and second-line regimens. Regular monitoring of antibiotic resistance patterns and timely implementation of enhanced infection prevention strategies are the need of the hour.

## Introduction

Neonatal sepsis, also known as sepsis neonatorum, is a life-threatening multisystem syndrome that manifests during a period of 28 days after birth (0–28 days of life) and is the outcome of bloodstream infections. It is classified as either early- or late-onset (EOS or LOS), with each form having a distinct etiology [1]. The former, EOS, caused by the transmission of pathogens from the maternal-genitourinary system or gastrointestinal flora to the neonates or fetus, is now controlled to a greater extent, primarily due to the introduction of universal screening of Group B streptococcus in pregnant women and intrapartum antibiotic prophylaxis. However the latter continuous to cause severe challenges to many countries and usually occurs when pathogens get transmitted from the surrounding environment, after delivery and also by a late manifestation of vertically transmitted infection [2]. In the absence of prompt diagnosis and effective treatment, the condition can worsen, resulting in serious complications involving organ failures, septic shock, and even death [3]. Neonatal sepsis is a leading global cause of disability and mortality, and it continues to pose a considerable diagnostic dilemma for neonatologists, exacerbated by non-specific clinical signs and diagnostic ambiguities in early stages [4].

Despite advances in neonatal medicine and care, the prevalence of severe sepsis has increased over the last two decades, particularly in developing countries [5]. For example, a recent systematic review and meta-analysis revealed that neonatal sepsis accounts for more than a fifth of a million deaths worldwide each year, out of which 40.98% occur in the African continent [6,7]. During the neonatal period, bacterial sepsis can originate from either maternal or nosocomial sources, and it encompasses a range of etiologies, including both Gram-positive (GPB) and Gram-negative bacteria (GNB) [8]; the latter group is predominant in sub-Saharan African countries [8]. In low- and lower-middle-income countries, GNB, which are associated with substantial antibiotic resistance, account for 60% of neonatal sepsis [9].

Risk factors associated with neonatal sepsis in sub-Saharan Africa can be maternal [10,11] or peripartum/delivery [11,12], as well as environmental [6,12]. Neonatal sepsis remains a substantial and growing burden in Ethiopia, with a pooled prevalence of 45% [13]. It is one of the three leading causes of neonatal mortality in the country, accounting for around one-third of all neonatal deaths [14].

In Ethiopia, sepsis management is often compromised due to limited awareness of warning signs, insufficient medical training, inadequate blood culture facilities, outdated antibiotic guidelines, and rapidly increasing drug resistance [15], and Dilla is no exception. Neonatal sepsis is one of the top ten reasons for referrals in the current study setting, with an average of sixty neonates getting admitted to the neonatal intensive care units and paediatric wards every month. The available options for managing neonatal sepsis at the current study setting are empirical therapy with first-line drugs (ampicillin and gentamicin) and subsequent treatment with second-line drugs (cloxacillin, ceftriaxone, and gentamicin) in severe cases. These empirical regimens are based on national guidelines; however, they have not been recently validated in regard to the susceptibility patterns of bacterial pathogens existing in the study area. In other words, the currently existing local antibiotic resistance data are obsolete to a greater extent. Due to changes in the spectrum of bacterial pathogens and antibiograms, updated and comprehensive data on local prevalence, aetiological profiles, antibiotic susceptibility, and associated factors are essential to guide clinicians in initiating an effective therapy. This study was to assess the bacterial profile and antibiotic susceptibility patterns of sepsis-causing pathogens among neonates at Dilla University Referral Hospital and also the associated factors.

## Materials and methods

### Study design, period, and study area

A cross-sectional study was conducted at Dilla University Referral Hospital (DURH) from August 1 to October 31, 2023. The study setting is located 85 kilometers away from Hawassa, the regional capital of the Southern Nations, Nationalities, and Peoples Region. The hospital provides preventive, curative, and rehabilitative care for outpatients and inpatients, as well as services from the pharmacy, maternal and paediatric healthcare unit, and laboratory department. The neonatal ward (both inpatient and outpatient) and the neonatal intensive care unit (NICU) are part of the paediatric department, which has an average of 300 outpatient department visits and NICU admissions during a span of three-month period. The paediatric department has a total of 45 staff members specialized in neonates, seven of whom are paediatricians. The unit has 40 cots and is equipped with 9 incubators and 13 phototherapy devices.

### Study population

The study population comprised all treatment-naïve neonates (of either sex, ≤ 28 days of age) admitted to the NICU, who met the inclusion and exclusion criteria outlined below.

### Inclusion criteria

All neonates (aged 0–28 days) admitted to the NICU and clinically suspected of having sepsis were diagnosed by paediatricians based on clinical criteria practiced in the hospital [16,17], and whose parents/caretakers volunteered to participate were included in the study, subjected to the following exclusion criteria.

### Exclusion criteria

Neonates born with gross congenital malformations or life-threatening anomalies, or any other metabolic disorders, were excluded. Those who were unable to provide samples, those with incomplete medical records, and those who were on antibiotics (exclusively the neonates, not the mother) or under follow-up at the time of sample collection were also excluded.

### Sample size determination

Epi-Info version 7.2 software was used to calculate the sample size based on a single population proportion formula. The following parameters were incorporated: a 95% confidence interval (z = 1.96), a 5% margin of error (d = 0.05), and a proportion of 21%, which was based on a previous study conducted in Saint Paul, Addis Ababa [18]. This calculation provided an initial sample size of 255.


$$\begin{array}{c} n=\frac{{\left(\frac{Z\alpha }{\text{2}}\right)}^{\text{2}}x\;p(\text{1}-p)}{{\left(d\right)}^{\text{2}}}=\frac{{\left(\text{1}.\text{9}\text{6}\right)}^{\text{2}}\times \text{0}.\text{2}\text{1}(\text{1}-\text{0}.\text{2}\text{1})\;}{{\left(\text{0}.\text{0}\text{5}\right)}^{\text{2}}}\;=\text{2}\text{5}\text{5} \end{array}$$

To accommodate a potential 5% non-response rate, the final sample size was increased to 280.

### Sampling technique

A systematic random sampling technique was used to select study participants who met the inclusion criteria, and the process was continued until the required sample size was attained. The sampling interval was calculated by dividing the total number of target patients by the sample size. The k^th^ value was inferred from the N/n formula (where ‘N’ is the number of neonates admitted to the hospital during the same period and ‘n’ is the sample size), which corresponded to a daily admission rate equalling two neonates during the study period. The first participant was selected through a lottery method, and subsequent members were recruited continuously.

### Outcome variables

The primary outcome of this study is the prevalence of culture-confirmed cases of neonatal sepsis, as defined by the isolation of bacteria from blood culture. The secondary outcomes are the bacterial profile and their antibiotic susceptibility patterns, as well as the identification of independent factors that influence the magnitude of neonatal sepsis.

### Independent variables of interest

Based on an extensive literature survey, sixteen variables suspected to be associated with neonatal sepsis were identified and included in the study. These variables broadly comprised maternal factors related to socio-demographic and obstetric data, as well as neonatal-related clinical data, and some environmental factors were also included.

### Data collection

The study was conducted via a questionnaire meant for parents and a review of the medical records following clinical assessments. Participants were included in the study after obtaining signed voluntary and informed consents from parents. The structured questionnaire was prepared based on prior studies [19–21]. Minor modifications were made in the questionnaire to fit in the current context. Well-trained health professionals conducted face-to-face interviews with mothers. Relevant socio-demographic details (such as age, sex, maternal education, occupation, residence, and monthly income) and obstetric and other maternal factors were meticulously incorporated into the datasheet.

### Data quality

To ensure data quality, 5% (n = 14) of the total sample size was pre-tested at the healthcare facility in the study area prior to the commencement of actual data collection. The data collectors were given one day of training by the Principal Investigator. Quality control measures were implemented throughout the laboratory work to ensure reliability of findings, and the data obtained were checked daily for completeness, accuracy, clarity, and consistency. Standard operating procedures were strictly followed for each operation; the expiry dates of the media and reagents and the quality control parameters were checked in accordance with CLSI guidelines. All culture media were prepared according to the manufacturer's instructions, and tested for sterility by incubating 5% of each batch at 35–37°C overnight to evaluate any possible contamination. After preparation, all culture plates and antibiotic discs were stored at the recommended temperature, 2–8°C. The reference strains of *S. aureus* (ATCC 25923), *E. coli* (ATCC 25922), and *P. aeruginosa* (ATCC 27853) were used, and *S. aureus* (ATCC 43300) was employed to validate the MRSA isolates. All the reference strains were obtained from the Ethiopian Public Health Institute.

### Blood sample collection, culture, and identification

Blood culture is considered the gold standard for confirming neonatal sepsis, as it can identify the causative pathogen directly. Two to three millilitres of blood were withdrawn twice aseptically using a butterfly vacutainer from two separate peripheral venipuncture sites (which were prepared with povidone–iodine and 70% alcohol) within an interval of 30–60 minutes. The withdrawn blood samples were inoculated directly into BD BACTEC™ culture vials and incubated aerobically at 37°C immediately afterwards. Routine inspections were performed twice daily for a week to detect any probable bacterial growth, the signs of which include uniform/subsurface turbidity, hemolysis, coagulation of the broth, surface floccular deposits, pellicle formation, and gas production. The samples were examined further by Gram staining and then sub-cultured aseptically onto blood agar, chocolate agar, MacConkey agar, and mannitol salt agar. The chocolate agar plates were incubated in a candle jar at 37°C with 5–10% CO_2_, while the other three agar plates (blood agar, MacConkey agar, and mannitol salt agar) were incubated aerobically for 18–24 h at 37°C [15].

Simultaneous growth in both vials was interpreted as positive, whereas growth in any one of the blood cultures was considered a probable contamination (pseudo bacteremia). Bacterial contaminants were defined as the growth of common skin flora (e.g., Micrococcus spp., Bacillus spp., other than *B. anthracis*, or coagulase-negative staphylococci (CoNS)) from a single culture vial without any corresponding clinical evidence of infection, and if found, were excluded from the final analysis of culture-positive sepsis. Blood culture showing no signs of bacterial growth was sub-cultured into the aforementioned pairs of agar media after a week, and was considered culture-negative if no growth occurred. Pure cultures of bacterial isolates were then identified and confirmed as specific species. The morphological, biochemical, and physiological characteristics of isolated bacteria were evaluated using laboratory methods [22]. In brief, GPB were identified by means of catalase, coagulase tests, Christie–Atkins–Munch-Petersen test, ability to ferment on mannitol salt agar, while GNB isolates were identified biochemically using a series of tests, viz., catalase, oxidase, indole, citrate, urease, H₂S production, methyl red, Voges–Proskauer, and triple-sugar iron.

### Antibiotic susceptibility testing

The antibiotic susceptibility profile was obtained using the Kirby-Bauer disc diffusion technique according to the criteria set by CLSI [23], as well as the hospital antibiotic policy [16,17]. Inocula of the respective bacteria were prepared in sterile normal saline to ensure an equivalent density with respect to the 0.5 McFarland standard. The test organisms were then uniformly swabbed onto Mueller–Hinton agar and exposed to a concentration gradient of antibiotic diffusion. The agar was then incubated at 37°C for 16–18 hours. Commercially available antibiotic discs (Oxoid, Basingstoke, Hampshire, UK) were used as per the CLSI guidelines. For the profile testing of GPB: ampicillin (10 µg), amoxicillin-clavulanate (20/10 µg), cefoxitin (30 µg), erythromycin (15 µg), chloramphenicol (30 µg), clindamycin (2 µg), ciprofloxacin (5 µg), tetracycline (30 µg), gentamicin (10 µg), and sulfamethoxazole-trimethoprim (1.25/23.75 µg). In the case of GNB, amoxicillin-clavulanate (30 µg), ceftriaxone (30 µg), cefotaxime (30 µg), ceftazidime (30 µg), ciprofloxacin (5 µg), chloramphenicol (30 µg), tetracycline (30 µg), gentamicin (10 µg), and sulfamethoxazole-trimethoprim (1.25/23.75 µg) were employed [24]. The diameters of the zones of inhibition were measured to the nearest millimeter and categorized as susceptible, intermediate, or resistant (CLSI guidelines). Isolates were classified as either susceptible or resistant to an antibiotic, and all isolates with intermediate resistance were considered as resistant to improve the fitness in subsequent statistical analyses; multi-drug resistance (MDR) was defined as the acquired inability to respond to at least one agent in three or more antibiotic categories [25]. An empirical antibiotic therapy was subsequently tailored according to the results of blood culture and susceptibility testing.

### Determination of MRSA and ESβL-producing GNB

The identification test for methicillin-resistant *Staphylococcus aureus* (MRSA) was performed in accordance with the CLSI criteria using the cefoxitin disc diffusion assay, and also by screening GNB isolates for ESβL production. The GNB isolates that were resistant to at least the third-generation cephalosporins (zones of inhibition ≤25 mm for ceftriaxone, ≤ 27 mm for cefotaxime, and/or ≤22 mm for ceftazidime) were considered as probable ESβL producers, and further phenotypic detection of ESβL-producing enterobacteria was performed using the double-disc synergy test. In brief, a lawn culture of the test isolate was prepared on Mueller-Hinton agar using a 0.5 McFarland standard suspension. A disc containing amoxicillin-clavulanic acid (20/10 µg) was placed in the centre of the plate, and discs containing the third-generation cephalosporins, ceftazidime (30 µg), cefotaxime (30 µg), and/or ceftriaxone (30 µg) were placed 20 mm (centre-to-centre) apart from the amoxicillin-clavulanic acid disc. After incubation for 16–18 hours at an ambient temperature of 35 ± 2°C, the test was considered indicative of ESβL production if an enhanced zone of inhibition was observed between any one of the cephalosporin antibiotic discs and the amoxycillin/clavulanic acid discs.

### Data processing and analysis

The collected data were coded, cleaned, and entered into Epi-Data version 4.2. They were then exported to SPSS for further analysis; IBM SPSS Statistics for Windows, version 25 (IBM Corp., Armonk, N.Y., USA) was used. Descriptive statistics, including frequencies, means, and standard deviations, were computed. The dependent variable in the study was the proportion of culture-confirmed cases of neonatal sepsis, with 95% confidence intervals (CIs). The susceptibility of bacteria to the tested antibiotics is presented as percentages. Multi-collinearity among variables was assessed using the multi-collinearity test (variance inflation factor and tolerance test). Bivariable and multivariable logistic regression analyses were performed to examine the association among variables and infections. In the former model, variables with *p*-values  ≤  0.25 were selected, whereas in the latter, *p*-values  ≤  0.05 were considered statistically significant.

### Ethical considerations

The study was approved by the Institutional Review Board of the College of Medicine and Health Sciences at Dilla University (Protocol unique no. DUIRB/037/23–17). Permission to conduct the study was obtained from the relevant management. In addition, written informed consents from parents/guardians were obtained before the collection of data and blood samples. The purpose of the study, including the benefits and risks, was clearly explained to the participants. They were also informed that their involvement was voluntary and that they could withdraw at any time without giving a reason. Information provided by patients was kept confidential, and specimens collected were analysed only for the intended purposes. Neonates found culture-positive were referred to pediatricians for proper orientation and treatment (in accordance with our NICU protocol), as well as follow-up.

## Results

### Socio-demographic characteristics

In this study, 280 blood samples were collected from neonates suspected of sepsis. The overall response rate was 100%. The majority of neonates (n = 164, 58.6%) were aged under 14 days; more than half of the neonates were females (n = 155, 55.4%). 50% (n = 142, 50.7%) rural dwellers; more than a quarter of parents (26.8%) were illiterate. The monthly family income of 49.3% were greater than 2000 Ethiopian birr. The socio-demographic characteristics are summarised in Table 1.

**Table 1:** Socio-demographic characteristics of neonates/parents (n = 280).

Variables	Category	Frequency	Percentage (%)
Sex	Male	125	44.6
	Female	155	55.4
Age group in days	<14	164	58.6
	14-28	116	41.4
Residence	Urban	138	49.3
	Rural	142	50.7
Educational status	Illiterate	75	26.8
	Primary	73	26.1
	Secondary	76	27.1
	College and above	56	20.0
Occupation	Farmer	81	28.9
	Homemakers	52	18.6
	Govt. employee	38	13.6
	Others	109	38.9
Monthly income in ETB	<1000	45	16.1
	1001-2000	112	40.0
	>2000	123	49.3

**Note:** ETB = Ethiopian Birr

### Clinical profiles

Of the entire neonates included in the study, around two-thirds (64%, n = 198) were delivered in hospital settings. More than half (54.3%, n = 152) of them had birth weight below 2.5 kg. Spontaneous vaginal delivery corresponded to 48.2% (n = 135), and delivery via an indwelling device accounted for 62.1% (n = 174); 52.9% (n = 148) of the neonates were born prematurely, i.e., before the completion of 37 weeks of gestation. Premature rupture of membrane was observed in 55.4% (n = 155) of cases, and preterm premature rupture of the membrane occurred in the case of 22.1% (n = 62) (Table 2).

**Table 2:** Clinical profile of study participants (n = 280).

Variables	Category	Frequency	Percentile
Place of delivery	Home	82	36.0
	Health facility	198	64.0
Weight of neonate at birth	≤2.5	152	54.3
	>2.5	128	45.7
Mode of delivery	Cesarean section	112	40.0
	Spontaneous vaginal	135	48.2
	Instrumental	33	11.8
Indwelling device during delivery	Yes	106	37.9
	No	174	62.1
Duration of hospitalization before delivery	≤12 h	22	7.8
	1-3 days	126	45.0
	4-10 days	117	41.8
	>10 days	15	5.4
Gestational age in weeks	≤ 37	148	52.9
	>37	132	47.1
Prolonged labor	Yes	219	78.2
	No	61	21.8
Premature rupture of the membrane	Yes	155	55.4
	No	125	44.6
Preterm premature rupture of membrane	Yes	62	22.1
	No	218	77.9
Parity	Primiparous	100	35.7
	Multiparous	180	64.3

### Prevalence of culture-positive neonatal sepsis

**Fig 1:**
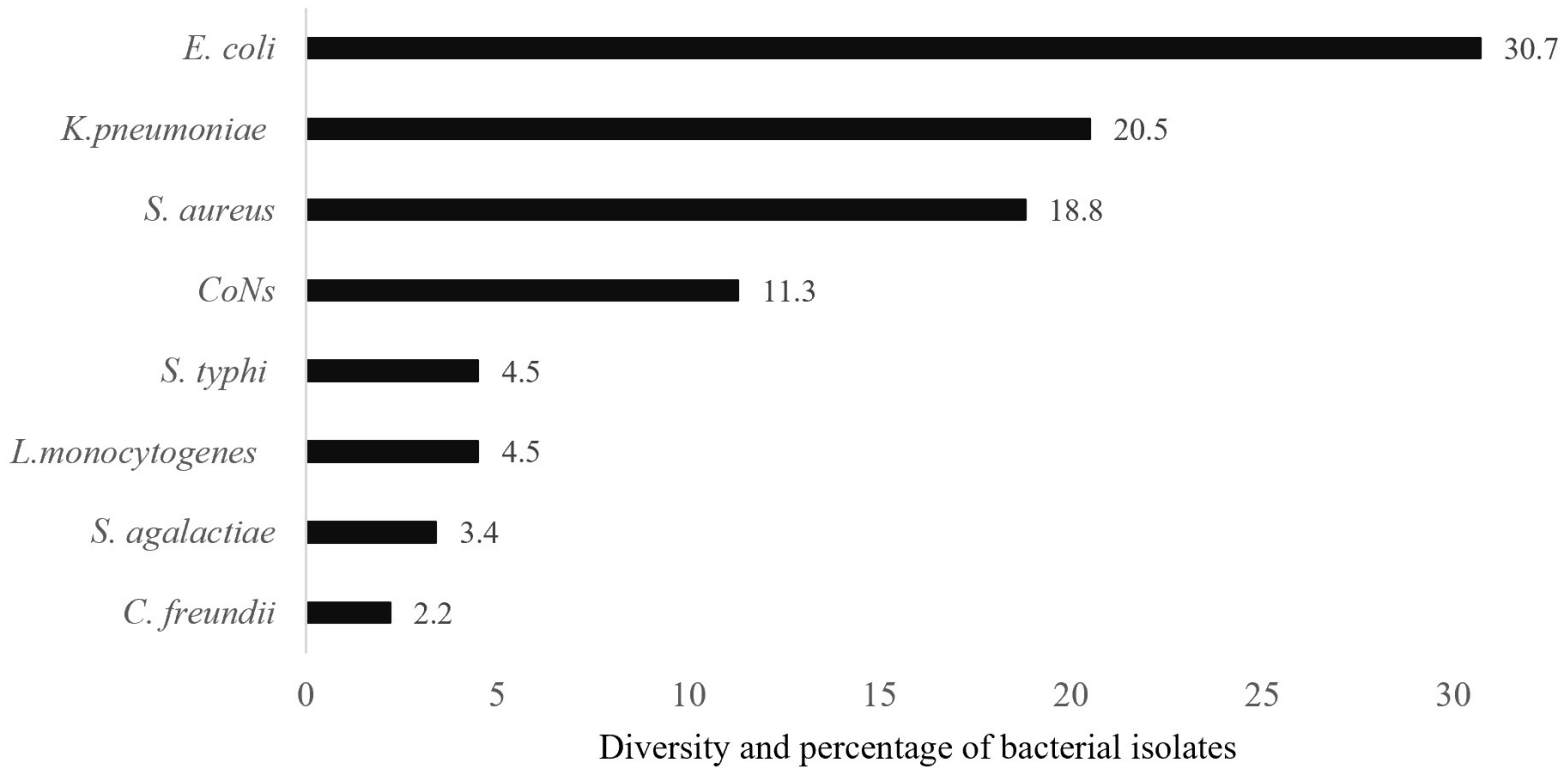
Diversity and percentage of bacterial isolates.

The overall prevalence of culture-confirmed cases of bacterial sepsis (n = 88) was 31.45% [95% CI: 26.0–37.2]. Of the total 280 blood samples collected from neonates clinically suspected of sepsis, 88 bacterial isolates representing nine distinct species were identified, out of which 57.9% (n = 51) were GNP, and 42% (n = 37) were GPC. The most frequently isolated pathogen was Escherichia coli, accounting for 30.7% (n = 27) of the entire isolates, followed by *Klebsiella pneumoniae,* 20.5% (n = 18), Staphylococcus aureus, 18.8% (n = 16), and coagulase-negative Staphylococcus (CoNS), 11.3% (n = 10). All other pathogens combined constituted less than 19.3% of the total cases. The least commonly isolated pathogens were Streptococcus agalactiae and *Citrobacter freundii*, which accounted for 3.4% (n = 3) and 2.2% (n = 2), respectively (Fig 1).

### Antibiotic susceptibility profiles of GNB

The susceptibility profiles of GNB (n = 51) against different antibiotics are presented in Table 3 as either susceptible or resistant. Intermediate results were also considered resistant in the statistical analysis, as previously mentioned.

**Table 3:** Antimicrobial susceptibility profiles of GNB.

Classes	Antibiotics	Patterns	GNB n (%)				Total	
			EC (n = 27)	KP (n = 18)	CF (n = 2)	ST (n = 4)		n = 51
Aminopenicillin	Ampicillin	S	0	NT		0		0
		R	27(100)	NT	2(100)	4(100)		33(100)
β-Lactam/β-lactamaseinhibitor combinations	Amoxicillin-clavulanate	S	0	1(5.6)	0	0		1(2)
		R	27(100)	17(94.4)	2(100)	4(100)		50(98)
Third generation of cephalosporins	Ceftriaxone	S	0	0	0	0		0
		R	27(100)	18(100)	2(100)	4(100)		51(100)
	Cefotaxime	S	3(10)	4(22.2)	0	0		7(13.7)
		R	24(90)	14(87.8)	2(100)	4(100)		44(86.3)
	Ceftazidime	S	4(14.8)	3(16.7)	1(50)	0		8(15.7)
		R	23(85.2)	15(83.3)	1(50)	4(100)		43(84.3)
Quinolones	Ciprofloxacin	S	22(81.5)	11(61.1)	2(100)	4(100)		39(76.5)
		R	5(18.5)	7(38.9)	0	0		12(23.5)
Amphenicol	Chloramphenicol	S	24(88.9)	18(100)	2(100)	4(100)		48(94.1)
		R	3(11.1)	0	0	0		3(5.9)
Tetracycline	Tetracycline	S	15(55.6)	10(55.6)	1(50)	NT		26(55.3)
		R	12(44.4)	8(44.4)	1(50)	NT		21(44.7)
Folate pathway inhibitors	Sulfamethoxazole-trimethoprim	S	14(51.9)	4(22.2)	1(50)	3(75)		22(43.1)
		R	13(48.3)	14(87.8)	1(50)	1(25)		29(56.9)
Aminoglycoside	Gentamicin	S	11(40.7)	13(72.2)	2(100)	NT		26(55.3)
		R	16(59.3)	5(27.8)	0	NT		21(44.7)

**n:** number of isolates, **S**: susceptible, **R**: resistant, **NT**: Not Tested, *NT corresponds to a change in the denominator (total number of isolates tested). EC: *E. coli*, KP: *K. pneumoniae*, CF: *C. freundii*, ST: S. Typhi

The GNB isolates showed 100% (n = 33) resistance to ampicillin, 98% (n = 50) to amoxicillin-clavulanate, 100% (n = 51) to ceftriaxone, and 84.3% (n = 43) to ceftazidime. However, most of these isolates showed relatively higher levels of susceptibility to chloramphenicol and ciprofloxacin, i.e., 94.1% (n = 48) and 76.5% (n = 39), respectively. The predominant species of GNB, *E. coli*, invariably showed 100% (n = 27) resistance to ampicillin, amoxicillin-clavulanate, and ceftriaxone. Conversely, they exhibited a significantly higher level of susceptibility, 88.9% (n = 24) to chloramphenicol and 81.5% (n = 22) to ciprofloxacin. The second most common isolate, *K. pneumoniae,* also showed a higher level of resistance, 100% (n = 18) to ceftriaxone, 94.4% (n = 17) to amoxicillin-clavulanate, and 87.8% each (n = 14) to cefotaxime and sulfamethoxazole-trimethoprim (Table 3). All the S. Typhi isolates were resistant to ampicillin, amoxicillin-clavulanate, and ceftriaxone (100%, n = 4). Similarly, the *C. freundii* isolates demonstrated very high resistance to ampicillin, amoxicillin-clavulanate, and ceftriaxone (100%, n = 2), but were 100% susceptible to chloramphenicol and gentamicin (n = 2).

### Antibiotic susceptibility profile of GPB

The susceptibility profiles of GPB isolates (n = 37) to different antibiotics are presented in Table 4 as either susceptible or resistant. They showed higher levels of resistance to erythromycin (70.3%, n = 26) and tetracycline (67.2%, n = 23). In contrast, these isolates were highly susceptible to chloramphenicol (100%, n = 37) and clindamycin (87.9%, n = 29), and also moderately susceptible to gentamicin (58.8%, n = 20). Analysis of species-specific resistance rates indicated that most of the *S. aureus* isolates were equally resistant to both sulfamethoxazole-trimethoprim and erythromycin (62.5%; n = 10) and also gentamicin (43.7%; n = 7), but to a slightly lower extent. On the other hand, 100% (n = 16) and 81.3% (n = 13) of them were susceptible to chloramphenicol and clindamycin, respectively. Among the sixteen isolates of *S. aureus*, 31.2% (n = 5) were MRSA.

**Table 4:** Antimicrobial susceptibility patterns of GPB.

Classes	Antibiotics	Patterns	GPB n (%)					Total % (n) (n = 37)
			*S. aureus* (n = 16)	CoNs (n = 10)	*S. agalactiae* (n = 3)	*E. faecalis* (n = 4)	*L. monocytogenes* (n = 4)	
Aminopenicillin	Ampicillin	S	NT	NT	0	1(25)	0	1(9)
		R	NT	NT	3(100)	3(75)	4(100)	10 (91)
β-Lactam/β-lactamaseinhibitor combinations	Amoxicillin-clavulanate	S	NT	NT	NT	NT	0	0
		R	NT	NT	NT	NT	4(100)	4(100)
Cephalosporin	Cefoxitin	S	11(68.8)	9(90)	NT	NT	NT	20(76.9)
		R	5 (31.2)	1(10)	NT	NT	NT	6(23.1)
Macrolides	Erythromycin	S	6 (37.5)	2(20)	3(100)	0	0	11(29.7)
		R	10(62.5)	8(80)	0	4(100)	4(100)	26(70.3)
Amphenicol	Chloramphenicol	S	16(100)	10(100)	3(100)	4(100)	4(100)	37(100)
		R	0	0	0	0	0	0
Lincosamide	Clindamycin	S	13(81.3)	10(100)	3(100)	NT	3(75)	29(87.9)
		R	3(18.7)	0	0	NT	1(25)	4(12.1)
Quinolones	Ciprofloxacin	S	11(68.8)	1 (10)	NT	3(75)	2(50)	17(50)
		R	5(31.2)	9 (90)	NT	1(25)	2(50)	17(50)
Tetracycline	Tetracycline	S	10(62.5)	0	3(100)	1(25)	0	14(37.8)
		R	6(37.5)	10(100)	0	3(75)	4(100)	23(67.2)
Folate pathway inhibitors	Sulfamethoxazole-trimethoprim	S	6 (37.5)	8 (80)	NT	2(50)	4(100)	20(58.8)
		R	10(62.5)	2(20)	NT	2(50)	0	14(41.2)
Aminoglycoside	Gentamicin	S	9(56.3)	6(60)	NT	3(75%)	2(50)	20(58.8)
		R	7(43.7)	4(40)	NT	1(25%)	2(50)	14(41.2)

**n:** number of isolates, **S**: susceptible, **R**: resistant, **NT**: Not Tested, *NT corresponds to a change in the denominator (total number of isolates tested).

In the current study, isolates of CoNs were resistant to tetracycline (100%, n = 10), ciprofloxacin (90%, n = 9), and erythromycin (80%, n = 8). Only 10% (n = 1) of CoNs were found to be methicillin-resistant. On the other hand, 100% (n = 10) isolates of CoNs were susceptible to both clindamycin and chloramphenicol, and 90% (n = 9) were susceptible to cefoxitin. The *E. faecalis* isolates obtained were highly resistant to erythromycin: 100% (n = 4) and also to both tetracycline and ampicillin, 75% (n = 3 each). All entire *L. monocytogenes* isolates tested were invariably resistant to ampicillin, amoxicillin-clavulanate, and erythromycin (100%, n = 4).

### Resistance to the first and second line regimens

It was found that 27.27% (n = 20) of the isolates demonstrated resistance to the first-line regimen (ampicillin-gentamicin), whereas 25% (n = 22) were resistant to the second-line regimen (gentamicin-ceftriaxone). Overall, nearly half of the isolates (47.7%) showed resistance to both regimens.

### Multi-drug Resistance Profile and ESβL-producing GNB

The prevalence of antimicrobial resistance associated with sepsis is exceptionally high, with a striking rate of 94.3% (n = 83) of bacteria being resistant to at least one of the antibiotics tested. Among the total 88 bacterial isolates, 70.5% (n = 62) were MDR, comprising 62.2% (n = 23) of the total of GPB and 74.5% (n = 38) of the GNB. Among the GPB, 43.8% (n = 7) of *S. aureus*, 90% (n = 9) of CoNs, 100% (n = 4) of *L. monocytogenes*, and 75% (n = 3) of *E. faecalis* were found to be MDR. In the case of GNB, MDR was shown by *E. coli* (74%, n = 20) and *K. pneumoniae* (88.9%, n = 16) (Table 5). Notably, 27.5% (n = 14) of GNB were phenotypically confirmed as ESβL producers. The most common ESβL producer was *E. coli* (15.7%, n = 8), followed by *K. pneumoniae* (9.8%, n = 5) as shown in Table 5. The detailed antibiogram is given in the S1 Table.

**Table 5:** Multiple antibiotic resistance profiles of pathogens.

Bacteria isolated	Classes of antibiotics n (%)			MDR n (%)	ESβL n (%)
	R_3_	R_4_	R_5_		
GPB (n = 37)					
*S. aureus*	2(12.5)	4(25)	3(18.8)	9(56.25)	NT
CoNs	5(50)	3(30)	1(10)	9(90)	NT
*E. faecalis*	2(50)	1(25)	0	3 (75)	NT
*S. agalactiae*	0	0	0	0	NT
*L. monocytogenes*	2(50)	0	2(50)	4(100)	NT
Total	11(29.7)	8(21.6)	6(16.2)	25(67.6)	
GNB (n = 51)					
*E. coli* (27)	11(40.7)	2(7.4)	7(25.9)	20(74.0)	8(15.7)
*K. pneumoniae* (n = 18)	4(22.2)	7(38.9)	5(27.8)	16(88.9)	5(9.8)
*C. freundii* (n = 2)	0	1(50)	0	1(50)	1 (2)
*S.* Typi (n = 4)	1(25)	0	0	1(25)	0
Total	16(31.4)	10(19.6)	12(23.5)	38(74.5)	14(27.5)
Cumulative total (n = 88)	27(30.7)	18(20.5)	18(20.5)	62(70.5)	

**n:** number of isolates, **R3:** resistant to three antibiotics, **R4:** resistant to four antibiotics, **R5:** resistant to five or more antibiotics. MDR: Multi-drug resistance, GPB: Gram-positive bacteria, GNB: Gram-negative bacteria, ESβL: Extended-spectrum beta-lactamase. *NT corresponds to a change in the denominator (total number of isolates tested).

### Association of independent variables with the prevalence of neonatal sepsis

Analytical aspects of the association among socio-demographic and clinical variables with respect to neonatal sepsis are summarized in Table 5. In bivariable logistic regression analysis, neonatal sepsis was found statistically significant with the locality of residence (*p*-value 0.04, COR: 2.15; 95% CI:1.83–3.68), place of delivery (*p*-value 0.079, COR: 1.52; 95% CI: 0.35–1.05), presence of indwelling device (*p*-value 0.19, COR: 1.4; 95% CI: 0.84–2.32), gestational age (*p*-value 0.007, COR:0.43; 95% CI: 0.23–0.79), length of hospital stays (*p*-value 0.001, COR: 4.72 95% CI: 1.82–12.27), prolonged labor (*p*-value 0.007, COR: 2.25 95% CI: 1.25–4.03), premature rupture of membranes (*p*-value 0.004, COR: 0.04; 95% CI: 0.26–0.77), parity (*p*-value 0.146, COR:0.66; 95% CI: 0.38–1.15), and preterm premature rupture of membrane (*p*-value 0.003, COR: 2.97; 95% CI: 1.46–6.04). In contrast, no significant associations were observed in regard to the age group and sex of the neonates, as well as the occupation of parents; therefore, these variables were excluded from further analysis.

Multivariable logistic regression analysis identified five independent predictors of neonatal sepsis. These include rural residence (*p*-value 0.015, AOR: 1.43; 95% CI: 1.22–5.84), caesarean section delivery (*p*-value 0.002, AOR: 3.23; 95% CI: 1.04–9.46), hospital stays exceeding ten days (*p*-value 0.001, AOR: 4.65; 95% CI: 1.55–13.9), prolonged labour (*p*-value 0.011, AOR: 3.35; 95% CI: 1.32–8.56), and preterm premature membrane rupture (*p*-value 0.001, AOR: 5.23; 95% CI: 4.11–9.49). These findings suggest that the identified factors significantly contribute to the risk of neonatal sepsis among the study population (Tables 6 and 7).

**Table 6:** Bivariate logistic regression analysis for blood culture positivity among neonates (n = 280).

Variables	Category	Bacterial isolates (%)	COR (95%CI)	*p*-value
Sex	Male	34 (47.6)	1.43 (0.85-2.39)	0.721
	Female	54(62.4)	1	
Age (days)	<14	41(46.6)	1.91 (1.14-3.18)	0.542
	14-28	46(53.4)	1	
Residence	Urban	32 (36.4)	1	
	Rural	56 (63.6)	2.15 (1.83-3.68)	**0.04**
Place of delivery	Home	32 (36.4)	1	
	Health facility	56 (63.6)	1.52 (0.35-1.05)	**0.079**
Birth weight (Kg)	≤2.5	50 (56.8)	0.86 (0.51-1.43)	0.565
	>2.5	38 (43.2)	1	
Ways of delivery	Cesarean section	39 (44.3)	4.28 (1.88-13.44)	**0.015**
	Instrumental	47 (53.4)	1.45 (0.59-1.69)	0.99
	Spontaneous vaginal	2 (2.3)	1	
Presence of an indwelling device	Yes	46 (52.3)	1.4(0.84-2.32)	**0.193**
	No	42 (47.7)	1	
Gestational age in weeks	<37	50 (56.8)	0.43 (0.23-0.79)	**0.007**
	≥37	38 (43.2)	1	
Duration of hospital stay after delivery	≤12 hours	11 (12.5)	1	
	1-3 days	22 (25.0)	1.54 (0.61-3.85)	0.352
	4-10 days	9 (10.2)	0.66 (.17-2.57)	0.553
	> 10 days	46 (52.3)	4.72 (1.82-12.27)	**0.001**
Prolonged labor	Yes	60 (68.2)	2.25 (1.25-4.03)	**0.007**
	No	28 (31.8)	1	
Premature rupture of the membrane	Yes	53 (60.2)	0.04 (0.26-0.77)	**0.004**
	No	35(39.8)	1	
Parity	Primiparous	26 (29.5)	0.66 (0.38-1.15)	**0.146**
	Multiparous	62 (70.5)	1	
Preterm premature membrane rupture	Yes	51 (57.9)	2.973(1.462-6.042)	**0.003**
	No	37 (42.1)	1	

**Table 7:** Multivariable logistic regression analyses of factors associated with blood culture positivity among neonates (n = 280).

Variables	Category	Bacterial isolates (%)	AOR (95% CI)	*p*-value
Residence	Urban	32 (36.4)		
	Rural	56 (63.6)	1.43 (1.22-5.84)	**0.015**
Ways of delivery	Caesarean section	39 (44.3)	3.23 (1.04-9.46)	**0.002**
	Instrumental	47 (53.4)		
	Spontaneous vaginal	2 (2.3)		
	More than ten days	46 (52.3)	4.65 (1.55-13.90)	**0.001**
Prolonged labor	Yes	60 (68.2)	3.36 (1.32-8.56)	**0.011**
	No	28 (31.8)		
	Multiparous	62 (70.5)		
Preterm premature membrane rupture	Yes	51 (57.9)	5.23 (4.11-9.49)	**0.001**
	No	37 (42.1)		

## Discussion

Neonatal sepsis is one of the major causes of referrals to the study setting; the overall prevalence of culture-confirmed cases of neonatal sepsis as per this study was 31.4% (95% CI: 26–37.2), indicating that the current study area is sepsis-prone, highlighting an urgent intervention. The higher prevalence of neonatal sepsis is often due to a combination of maternal and neonatal risk factors, such as unhygienic practices during delivery and postnatal care, and challenges within healthcare settings, particularly in resource-limited environments. The overall prevalence found in our study is at par with the values reported from a series of studies done in different Ethiopian cities (ranging from 33 to 36%), corresponding to varying bacterial etiology [26,27], These consistencies extend to the works done in other African countries, with comparable results from Ghana (29%) [28], and Sudan (31%) [29]. The observed similarities in the prevalence of neonatal sepsis across Sub-Saharan Africa can be attributed to some sort of common environmental, socio-economic, and health factors prevailing among demographically similar populations.

The prevalence of neonatal sepsis and the etiological agents found by us are different from those published in recent literature. For instance, recent studies carried out in other parts of the country showed a higher prevalence range, 40.93–45% [30,31], as well as from Tanzania (45%) [32], and Uganda (59%) [33]. Conversely, the prevalence found in our study exceeded that reported from Addis Ababa (21%) [18] and South Africa (20.5%) [34]. The prevalence of neonatal sepsis can fluctuate widely due to methodological inconsistencies across studies (e.g., diagnostic criteria), diversity in healthcare infrastructure involved, and the quality of intensive care provided. Furthermore, diverse population-associated risk factors, variations in sampling and culture techniques, study design, and sample size contribute much to these disparities.

Differences in antimicrobial resistance surveillance and infection control practices can also play crucial roles in explaining the observed alterations in prevalence rates. Gram-negative bacilli were the predominant and leading causes of neonatal sepsis in the study area, which agrees well with the outcome of studies done in other cities of Ethiopia [18, 26, 31, 35] and South Africa [34]. The predominance of GNB in neonatal sepsis can be attributed to peripartum transmission, hospital-acquired infections, intestinal and skin colonization, and suboptimal environmental hygiene.

On the contrary, studies done in Ghana [36] and Tanzania [25] reported that GPB were the common causes of neonatal sepsis. The bacterial isolates found in this study have previously been recognized as potential pathogens responsible for neonatal sepsis [8]; *E. coli* and *K. pneumoniae* were the most common organisms isolated, accounting for 51.1% of the cases. Isolates of *K. pneumoniae* frequently emerge as the leading cause of neonatal sepsis in Ethiopia [18,26,35,37] and Ghana [28], often showing significantly higher percentages than *E. coli*. However, the predominant one found in our study was *E. coli,* and it supports the notion that sepsis can further provide an opportunity for *E. coli* (K1 strains) to disseminate, eventually invading the central nervous system and causing neonatal meningitis. In the case of GPB, *S. aureus* and CoNs put together represent 29.5% of cases. *Staphylococcus aureus* was the single most common isolate, both in the case of early-onset and late-onset sepsis [38]. Coagulase-negative Staphylococci are also very common and are sometimes reported as the single most frequent isolate, especially in studies from Hawassa [39] and Tanzania [25].

Gram-negative bacteria are commonly associated with the maternal genitourinary tract and are frequently implicated in peripartum and nosocomial infections due to their ability to colonize mucosal surfaces and survive in hospital environments. In contrast, Gram-positive organisms like Group B *Streptococcus* and *S. aureus* are often more prevalent in community settings, where transmission is less influenced by hospital interventions and more by community carriage. Additionally, the widespread use of invasive procedures, antibiotic prophylaxis, and prolonged hospital stays in certain settings may select for GNB, while differing infection control practices and community health profiles can favour Gram-positive dominance [2].

In this study, GNB showed higher degrees of resistance to ampicillin (100%), amoxicillin-clavulanate (almost 100%), ceftriaxone (100%), cefotaxime (>80%), and ceftazidime (>80%), nullifying their empirical usage in suspected cases of neonatal sepsis in our study setting. Multiple studies from Ethiopia corroborate the extremely high resistance rates shown against ampicillin and the third-generation cephalosporins [26,27,31]. Moderately high resistance to extreme levels of resistance to amoxicillin-clavulanate was also reflected in other Ethiopian studies, i.e., between 57% and 100%, shown by various GNB species [27]. A systematic review of GNB neonatal sepsis in low- and lower-middle-income countries, including African nations, brings out the significant rates of resistance to ampicillin and third-generation cephalosporins (57% to 81% for ceftriaxone) [9].

The GPB exhibits higher levels of resistance to ampicillin, amoxicillin-clavulanate, erythromycin, and tetracycline. A systematic review and meta-analysis on paediatric sepsis in Ethiopia also indicates higher resistance to erythromycin and tetracycline by common *S. aureus* and coagulase-negative Staphylococci [40]. In the present study, over a quarter of *S. aureus* were MRSA (WHO prioritized pathogen), which resembles the trend in other African countries, where one-third of *S. aureus* isolates were MRSA [41]

The overall percentage of MDR in our study was 70.5%, which is congruent with the data described in a previous systematic review (78.86%) on Ethiopian neonatal sepsis [40]. Also, studies done in Hawassa and Jimma reported MDR rates of 78.3% and 88.4%, respectively [26,31]; *Staphylococcus aureus* was found to have the highest MDR rate as per a Tanzanian study [25], and our results are in tandem with this outcome. Nevertheless, *E. coli* remains a notable MDR GNB in neonatal sepsis in our study setting, and also in another study [18]; *K. pneumoniae* was the predominant MDR in certain earlier Ethiopian studies on neonatal sepsis [18,31,35].

WHO prioritized ESβL-producing GNB as a major public health menace among neonatal populations across Africa, including Ethiopia [31]. In the present study, over a quarter of the GNB isolates were ESβL producers. However, a study done in Tanzania specifically focusing on ESβL-producing Enterobacteriaceae in neonatal sepsis detected a lower prevalence of 10% only [42]. This variability in the prevalence of ESβL producers observed highlights the importance of updated local epidemiological data. Previous studies from Ethiopia corroborated that resistance to the WHO-recommended first-line regimen (ampicillin and gentamicin) is extremely severe, often necessitating reconsideration of its empirical use [18,31,43]. In the current study setting, ampicillin-gentamicin and gentamicin-ceftriaxone, respectively, are recommended as the first- and second line of drugs to treat neonatal sepsis. It is worrisome that most of the isolates showed considerable resistance against gentamicin (>38%), ampicillin (>48%), and ceftriaxone (> 60%), restricting their empirical usage. More than a quarter of isolates were resistant to the first-line regimen (ampicillin-gentamicin) and also the second-line regimen (gentamicin-ceftriaxone); WHO recommended a threshold of 5% resistance for a compulsory change in the empirical therapy in neonatal sepsis [44], and this should be taken seriously in the present context. Nearly half of the isolates demonstrated resistance to the combined first- and second-line regimens, highlighting the complexity and urgency involved in the effective management. Our results can be used as a benchmark in fixing the regimen and bringing in amendments in the local empirical antimicrobial therapy, whenever needed.

In this study, neonates whose parents live in rural areas have 1.43 times enhanced chance of developing sepsis compared to their counterparts, and this was similar to the trend found in an earlier Ethiopian study [45]. Neonates hospitalized for ≥10 days were about 4.65 times more at risk of developing sepsis, and this is in agreement with a couple of previous studies [27,46]. A longer duration of hospitalization inherently means extended contact with potential sources of infection, including antibiotic-resistant bacteria.

In our study, the rate of sepsis observed was higher in the case of those delivered by Cesarean section than for those corresponding to normal delivery. The former group may have a higher rate of sepsis due to the lack of exposure to the maternal vaginal microbiome, which is crucial for immune system development and protection against pathogens [47]. An earlier work in Ethiopia reported that Caesarean-delivered neonates were approximately three times more likely to develop sepsis [39]. Another vital factor that was significantly associated with the prevalence of neonatal sepsis was prolonged labor and preterm premature membrane rupture. A meta-analysis on maternal and neonatal sepsis done in sub-Saharan Africa identified prolonged labour as a factor, increasing the risk of neonatal sepsis considerably [11].

Along with empirical antibiotic therapy, supportive care and strict and close monitoring in the neonatal ICU are utmost important. The effective prevention involves strategies focused on the improvement of maternal health (consisting of prepartum prophylaxis and treating GBS during pregnancy), implementing rigorous infection control (maintaining hand hygiene and aseptic techniques in neonatal care settings) through screening protocols and early detection. Also, maternal and caregiver education are of great significance in neonatal sepsis management in resource-limited health care units like the current study setting.

The current study possesses several strengths in regard to the applicability of its findings. It was done to investigate the factors associated with neonatal sepsis, which have not been previously documented in the studied setting. The use of a multivariable logistic regression model enabled the adjustment of potential confounding variables, providing a more accurate assessment of factors associated with neonatal sepsis. Incorporating clinically relevant variables, such as Caesarean section delivery, hospital stays exceeding ten days, prolonged labour, and preterm premature membrane rupture, can enhance the accuracy of analyses, ensuring that the present set of results is relevant to clinicians. The study reveals valuable information on neonatal sepsis, which can substantiate the planning of future antibiotic stewardship programs.

### Limitations

Our study has several limitations, including its single-center design. Early and late onset sepsis were not differentiated. The only aerobic culturing would have limited the identification of anaerobic pathogens, *per se*. Serotyping of certain isolates was not done; antibiotics such as cloxacillin and vancomycin were not tested due to unavailability. Besides, isolated bacterial pathogens were not subjected to molecular diagnosis due to limited laboratory infrastructure. A further limitation of this study is the classification of intermediate results as resistant. Data on maternal prepartum or intrapartum antibiotics were not considered, which is also a limitation. Although this approach aligns with the clinical decision-making process, it may affect the generalizability and interpretation of overall results.

## Conclusion

This is the first report on the prevalence of neonatal sepsis in Dilla, revealing a moderately high overall prevalence of 31.4% with notable associations existing among maternal risk factors. Of the entire GNB, *E. coli,* and among GPB, *S. aureus* were found to be the most prevalent causative agents. There is an abrupt growth in antibiotic resistance shown by bacterial pathogens to several clinically relevant antimicrobials (such as ampicillin (100%), amoxicillin-clavulanate (almost 100%), ceftriaxone (100%), cefotaxime (>80%), and ceftazidime (>80%)) in the study setting. A substantial proportion (>90%) of the bacteria demonstrated resistance to at least one of the antibiotics tested. A startling finding of our study is that 70.2% of bacteria were MDR; WHO prioritized superbugs such as ESβL and MRSA were also detected. More than a quarter of isolates were resistant to the first- and second-line regimens. Strengthening the antimicrobial stewardship programs in healthcare facilities by implementing evidence-based prescription guidelines for neonatal sepsis is extremely important. Ensuring prompt initiation of appropriate empirical therapy based on updated resistance patterns is essential to minimize the aggravation of drug resistance. Significant predictors of neonatal sepsis in the study area are dwelling in rural areas, mode of delivery, prolonged hospital stays, prolonged labour, and preterm premature rupture of membranes. It is important to stress that the important findings from this study can be taken seriously by the policy makers, clinicians, and health workers.

## Supporting information

S1 Data Raw data. (XLSX)

S1 Table Antibiogram of bacterial isolates. (DOCX)
