# Supplementary material for: Sepsis neonatorum: Bacterial profile, antimicrobial resistance patterns, among neonates and associated factors, Dilla University Referral Hospital, southern Ethiopia, with special reference to WHO prioritized pathogens
Source: PLoS One. 2026 Jul 10;21(7):e0352190. doi: 10.1371/journal.pone.0352190 (PMC13353990; doi:10.1371/journal.pone.0352190)
Supplement: S1 Table — (DOCX) [file pone.0352190.s002.docx]

| Bacterial isolates | Antibiogram patterns | No of isolates  n (%) |
| --- | --- | --- |
| *E.coli* (n=20) | AMP, AUG, COT | 4 |
|  | AUG, AMP, COT | 3 |
|  | AUG, COT, TET | 2 |
|  | AUG, COT, GEN | 2 |
|  | AUG, TET, CTX, COT | 1 |
|  | AUG, TET, COT, CTX | 1 |
|  | AMP, AUG, COT, CIP, TET, CAZ | 3 |
|  | AUG, COT, TET, CIP, GEN, CH | 4 |
| *K.* *pneumoniae* (n=16) | AUG, CTX, COT | 4 |
|  | AUG, CIP, CTX, COT, TET | 3 |
|  | AUG, COT, TET, CAZ | 4 |
|  | AUG, CIP, TET, CAZ, COT | 3 |
|  | AUG, CIP, TET, COT, CTX | 2 |
| *C. freundii*(n=1) | AUG, TET, CAZ, COT | 1 |
| *S. Typi* (n=1) | AUG, CTX, COT | 1 |
| *S. aureus* (n=9) | TET, ERY, GEN | 1 |
|  | CIP, GEN, COT | 1 |
|  | TET, ERY, COT, CLI | 1 |
|  | TET, FOX, COT, CLI | 2 |
|  | TET, GEN, COT, CLI | 1 |
|  | CIP, TET, GEN, COT, ERY, CLI | 1 |
|  | FOX, TET,GEN, COT, ERY, CLI | 2 |
| *E. faecalis* (n=3) | CIP, TET, ERY, AMP | 1 |
|  | AMP, GEN, ERY | 1 |
|  | AMP, TET, ERY | 1 |
| *L. monocytogenes* (n=4) | AUG, AMP, TET | 1 |
|  | AMP, AUG, ERY | 1 |
|  | AMP, AUG, CIP, TET, ERY, GEN | 2 |

Supplementary Table 1: Antibiogram of bacterial isolates

AMP: Ampicillin; AUG: Amoxicillin/Clavulanic acid; COT: Cotrimoxazole (Trimethoprim/Sulfamethoxazole); TET: Tetracycline; GEN: Gentamicin; CTX: Cefotaxime; CIP: Ciprofloxacin; CAZ: Ceftazidime; ERY: Erythromycin; CLI: Clindamycin; FOX: Cefoxitin CH: Chloramphenicol
